# Determining the diet of wild Asian elephants (*Elephasmaximus*) at human–elephant conflict areas in Peninsular Malaysia using DNA metabarcoding

**DOI:** 10.3897/BDJ.10.e89752

**Published:** 2022-10-20

**Authors:** Nor Hafisa Syafina Mohd-Radzi, Kayal Vizi Karuppannan, Nurfatiha Akmal Fawwazah Abdullah-Fauzi, Abd Rahman Mohd-Ridwan, Nursyuhada Othman, Abdul-Latiff Muhammad Abu Bakar, Millawati Gani, Mohd Firdaus Ariff Abdul-Razak, Badrul Munir Md-Zain

**Affiliations:** 1 Department of Biological Sciences and Biotechnology, Faculty of Science and Technology, Universiti Kebangsaan Malaysia, 43000, Bangi, Selangor, Malaysia Department of Biological Sciences and Biotechnology, Faculty of Science and Technology, Universiti Kebangsaan Malaysia 43000, Bangi, Selangor Malaysia; 2 Department of Wildlife and National Parks (PERHILITAN), KM 10 Jalan Cheras, 56100, Kuala Lumpur, Malaysia Department of Wildlife and National Parks (PERHILITAN), KM 10 Jalan Cheras 56100, Kuala Lumpur Malaysia; 3 Centre for Pre-University Studies, Universiti Malaysia Sarawak, 94300 Kota Samarahan, Sarawak, Malaysia Centre for Pre-University Studies, Universiti Malaysia Sarawak 94300 Kota Samarahan, Sarawak Malaysia; 4 Faculty of Applied Sciences and Technology, Universiti Tun Hussein Onn Malaysia (Pagoh Campus), 84600 Johor, Malaysia Faculty of Applied Sciences and Technology, Universiti Tun Hussein Onn Malaysia (Pagoh Campus) 84600 Johor Malaysia; 5 Oasis Integrated Group (OIG), Institute for Integrated Engineering (I2E), Universiti Tun Hussein Onn Malaysia, 86400 Parit Raja, Johor, Malaysia Oasis Integrated Group (OIG), Institute for Integrated Engineering (I2E), Universiti Tun Hussein Onn Malaysia 86400 Parit Raja, Johor Malaysia

**Keywords:** Asian elephant, diet, rbcL, DNA metabarcoding, next-generation sequencing

## Abstract

Human–elephant conflict (HEC) contributes to the increasing death of Asian elephants due to road accidents, retaliatory killings and fatal infections from being trapped in snares. Understanding the diet of elephants throughout Peninsular Malaysia remains crucial to improve their habitat quality and reduce scenarios of HEC. DNA metabarcoding allows investigating the diet of animals without direct observation, especially in risky conflict areas. The aim of this study was to determine: i) the diet of wild Asian elephants from HEC areas in Peninsular Malaysia using DNA metabarcoding and ii) the influence of distinct environmental parameters at HEC locations on their feeding patterns. DNA was extracted from 39 faecal samples and pooled into 12 groups representing the different sample locations: Kuala Koh, Kenyir, Ulu Muda, Sira Batu, Kupang-Grik, Bumbun Tahan, Belum-Temengor, Grik, Kampung Pagi, Kampung Kuala Balah, Aring 10 and the National Elephant Conservation Centre, which served as a positive control for this study. DNA amplification and sequencing targeted the ribulose-bisphosphate carboxylase gene using the next-generation sequencing Illumina iSeq100 platform. Overall, we identified 35 orders, 88 families, 196 genera and 237 species of plants in the diet of the Asian elephants at HEC hotspots. *Ficus* (Moraceae), *Curcuma* (Zingiberaceae), *Phoenix* (Arecaceae), *Maackia* (Fabaceae), *Garcinia* (Clusiaceae) and *Dichapetalum* (Dichapetalaceae) were the highly abundant dietary plants. The plants successfully identified in this study could be used by the Department of Wildlife and National Parks (PERHILITAN) to create buffer zones by planting the recommended dietary plants around HEC locations and trails of elephants within Central Forest Spine (CFS) landscape.

## Introduction

Asian elephants (*Elephasmaximus*) are charismatic animals that have been categorised as an endangered species by the International Union for Conservation of Nature Red List (Williams et al. 2020) and listed under Appendix I of the Convention on International Trade in Endangered Species of Wild Fauna and Flora (CITES 2020). In Peninsular Malaysia, they are protected under the Wildlife Conservation Act 2010 (Act 716). From biodiversity inventories and dung count surveys, the wild population of *E.maximus* in Peninsular Malaysia is estimated at 1223–1677 individuals (Saaban et al. 2011), which are distributed across six states: Pahang, Terengganu, Kelantan, Kedah, Perak and Johor (Karuppannan et al. 2020). Karuppannan (2020) stated that Asian elephants travel within the Main Range Forest Complex spanning from southern Thailand to southern Peninsular Malaysia. The Main Range Forest Complex or Central Forest Spine (CFS) contains the Managed Elephant Ranges (MERs) which include three major population centres: the Belum-Temenggor complex, the Taman Negara National Parks and the Endau-Rompin Forest Complex (Saaban et al. 2011). Currently, the Asian elephants’ populations live in abundance within the MERs forest complexes (DTCP 2009, Jambari et al. 2019, Saaban et al. 2020, Karuppannan et al. 2020).

*E.maximus* are primarily threatened by the loss, fragmentation and degradation of habitat and poaching for ivory, skin, meat and leather (Choudhury et al. 2008). Urbanisation, agriculture, roads and human settlements have isolated the forest complexes. The Central Forest Spine Master Plan was introduced by the Malaysian Government to restore and maintain the connectivity of fragmented forests in Peninsular Malaysia (DTCP 2009). However, it needs to be updated with the latest evidence on the distribution of elephants and other wildlife (Saaban et al. 2020). Human–elephant conflict (HEC) occurs when the elephants’ natural habitat continues to shrink while their home range extends and overlaps with human settlements or cultivated areas. HEC cases include crop raiding, property damage, poisoning and injuries or deaths to humans and elephants (Zafir et al. 2016). *E.maximus* have been increasingly reported in areas, such as highways, rubber plantations, oil palm plantations, logged forests and human settlements (Campos-Arceiz 2013, Yamamoto-Ebina et al. 2016). Elephants are edge-specialists (Campos-Arceiz 2013), which causes them to be attracted to roadsides or highways. According to Bahar et al. (2018), *E.maximus* are prone to enter secondary forests beyond Protected Areas (PA) due to lack of connectivity between forests to critical corridor and linkages within the CFS. Elephants encroaching into small-scale village farms of rubber, oil palm and other plantation crops results in large financial losses for plantation owners (Zafir et al. 2016). The National Elephant Conservation Action Plan (NECAP) has been formed to guide all the conservation plans for *E.maximus* (PERHILITAN 2013) with central priority to improve the habitat quality in the wild, strengthen the enforcement of elephant-related laws and effectively manage HECs.

Asian elephants are “mega-gardeners” of the Malaysian tropical rainforests (Campos-Arceiz and Blake 2011), effectively dispersing seeds and seeds passing through mammalian guts have a greater chance of germinating (Sukumar 2003, Campos-Arceiz and Blake 2011). Being the largest terrestrial herbivore, *E.maximus* consume a wide range of foods to sustain their nutritional requirements. Elephants may feed for 14–19 hours a day, which amounts to 150 kg of food (Vancuylenberg 1977). *E.maximus* are generalised feeders that feed on more than 400 different plant species (Dutton 2008); the variation in choices is primarily influenced by the habitat and the food season (Olson 2002). Koirala et al. (2018) stated that nutrient composition in the diet of elephants varies by their sex and age. However, Swit (2016) acknowledged that *E.maximus* can be specialised when feeding on preferred plants. According to Olson (2002), they tend to include a higher proportion of dry matter like grass in their diet, which they often prefer along with monocotyledonous plants (English et al. 2014). Yamamoto-Ebina et al. (2016) discovered that non-grass monocotyledonous plants are favoured by elephants in the primary and logged forest habitats. Elephants also consume a range of fleshy fruits like mangoes (*Mangiferaindica*), jackfruits (*Artocarpusheterophyllus*), Ceylon olives (*Elaeocarpusserratus*), wild guavas (*Careyaarborea*), Java plums (*Syzygiumcumini*) and star apples (*Chrysophyllumroxburghii*) (Jothish 2013).

Using genomics tools, the diet of elephants can be studied from the perspective of conservation. Metagenomics is the combination of next-generation sequencing (NGS) and DNA barcoding (Mohd-Yusof et al. 2022) and it can effectively mine large datasets to identify diets, parasites and microbiota in the samples (Eisen 2007). Metabarcoding detects plants present in the diet and is a powerful tool to monitor ecosystems in terms of the degradation of species habitats. The rbcL and trnL locus regions have widely been used to investigate the diet of herbivores and omnivores (Poinar et al. 1998, Bradley et al. 2007, Hawlitschek et al. 2018, Mallot et al. 2018). According to previous studies, the best non-invasive sampling approach is collecting fecal samples as it does not require handling or observing the animals (Aifat et al. 2016, Abdul-Latiff et al. 2017, Karuppannan et al. 2019). In this study, the rbcL region was targeted to analyse the diet of *E.maximus*. Identifying the plant taxa preferred by wild *E.maximus* in HEC areas with environmental influences is crucial for HEC management.

With HEC incidents increasing yearly (PERHILITAN 2015), a better understanding of the dietary plants in HEC areas is required for effective conservation strategies. In Malaysia, studies utilising metabarcoding to detect the feeding habits of *E.maximus* are limited. Continuous, non-invasive sampling of elephant feces and long-term dietary observations from stored samples are critical to create the complete *E.maximus* feeding database. This study aims to determine the diet of wild Asian elephants in HEC hotspots throughout Peninsular Malaysia via DNA metabarcoding. We also investigate the influence of environmental parameters in HEC areas on the feeding habits of free-ranging *E.maximus*. The plant metabarcoding database generated from this study can be used by the Department of Wildlife and National Parks (PERHILITAN) in the national habitat enrichment programs to restore vast tracts of uninterrupted forests as elephant habitat within the CFS landscape (PERHILITAN 2013). Knowledge of dietary plant genera is useful to create buffer zones and subsequently reduce impacts of HEC.

## Material and methods

**1) Feacal Sampling**

**Figure 1.**
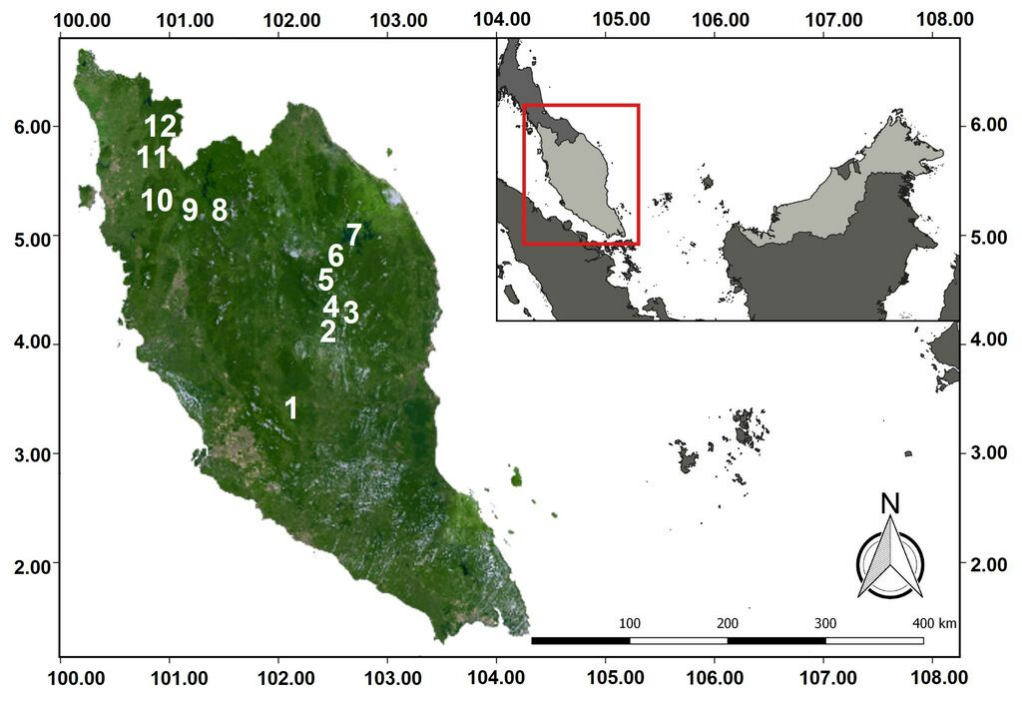
Map of Peninsular Malaysia with a close-up of various locations, where this study took place (1 = National Elephant Conservation Centre, 2 = Bumbun Tahan, 3 = Aring 10, 4 = Kg. Kuala Balah, 5 = Kg. Pagi, 6 = Kuala Koh, 7 = Kenyir, 8 = Kupang-Grik, 9 = Belum-Temenggor, 10 = Grik, 11 = Ulu Muda, 12 = Sira Batu).

Feacal samples of *E.maximus* were provided by the Wildlife Genetic Resource Bank (WGRB) of PERHILITAN, who collected them from various localities in Peninsular Malaysia based on HEC complaints that were lodged by the public (Fig. 1). All samples from Kuala Koh, Kenyir, Ulu Muda and Belum-Temenggor were collected by the Management and Ecology of Malaysian Elephants (MEME), University of Nottingham Malaysia. Samples contributed by MEME were also obtained from HEC locations, such as logged forests, non-logged forests, highways, human settlements, near human settlements etc. Field sampling was conducted based on the non-invasive sampling protocol for fresh feacal samples where an adequate amount of inner part of the elephant’s feces is scooped into individual feces or vial tube with complete sample details of sample ID, date and locality. Raw samples were immediately kept in a styrofoam box filled with ice packs before being transferred to a -20℃ freezer at the National Wildlife Forensic Laboratory (NWFL) of PERHILITAN. Captive samples from the National Elephant Conservation Centre (NECC) in Kuala Gandah, Pahang served as positive controls and baseline data for the diet of *E.maximus*.

**2) DNA extraction and amplification**

Laboratory work was performed at NWFL, PERHILITAN. Approximately 150 mg of the feacal sample was subjected to DNA extraction using the Qiagen QIAamp Fast DNA Stool mini kit (Qiagen, Germany). The extracted DNA was quantified spectrophotometrically on an Implen Nanophotometer. In this study, the 39 samples were pooled into 11 different DNA extracts corresponding to the different HEC localities with distinct environmental parameters, with one additional pooled sample representing the positive control (captivity) (Karuppannan 2020).

Polymerase chain reaction (PCR) for Illumina sequencing was performed twice. The first PCR was to amplify the targeted region of the ribulose-bisphosphate carboxylase (rbcL) gene; the second PCR was to index the purified PCR products. The gene was amplified using the forward primer rbcLZ1: 5’-TCGTCGGCAGCGTCAGATGTGTATAAGAGACAGATGTCACCACAAACAGAGACTAAAGCAAGT-3’ and the reverse primer rbcL19b: 5’-GTCTCGTGGGCTCGGAGATGTGTATAAGAGACAGCTTCTTCAGGTGGAACTCCAG-3’ (Poinar et al. 1998) with Illumina adapter overhang sequences. The PCR reaction contained Promega GoTaq Green Master Mix (10 µl), forward and reverse primers (1 µl, 10 µM each), nuclease-free water (6 μl), and DNA (2 µl) to give a final reaction volume of 20 µl. The reaction was performed on the Bio-Rad T100 Thermal Cycler using the following parameters: 94°C for 5 min; 40 cycles of 92°C for 15 sec, 57°C for 1 min and 72°C for 1 min; 72°C for 10 min. The PCR products were visualised using gel electrophoresis with 1% agarose gels in 1x TAE buffer to measure the size of amplicon. After gel visualisation, the first PCR showed bands for all samples with an amplicon size up to 157 bp (Bradley et al. 2007).

**3) Library construction for NGS**

The first PCR products were sent to GeneSeq Sdn. Bhd for library preparation and sequencing. They were purified using solid phase reversible immobilisation beads (Osman et al. 2020) and purified products underwent a second PCR to integrate Illumina dual index barcodes. The barcoded samples were pooled and purified. The indexed amplicons were quantified using a Denovix dsDNA High Sensitivity Assay. After normalising the concentrations of samples, the indexed amplicons were pooled into a single library for sequencing. The final library pool containing indexed amplicons were paired-end sequenced at 2 x 150 bp on the Illumina iSeq100 platform (Illumina Inc., USA).

**4) Bioinformatics and metabarcoding analysis**

The quality filtering and demultiplexing of sequences were performed using the CLC Genomic Workbench software (CLC) (Qiagen, USA) at the Evolutionary and Conservation Genetic Laboratory of the Department of Technology and Natural Resources, Universiti Tun Hussein Onn Malaysia. Quality scores were initially assessed across the Illumina data using FASTQ files. The operational taxonomical units (OTUs) were clustered at 97% similarity and represented by a single sequence. Rarefaction curves were plotted with the number of OTUs observed at a given sequencing depth using CLC. The plant genus classification of the OTUs was performed against an rbcL plant database with a confidence threshold of 97%. Using PAST 4.02 software, the alpha diversity indices of Shannon and Chao-1 index estimators measured the plant species richness in the elephants’ diet. The relationship between the samples was established using principal coordinate analysis (PCoA) in PAST 4.02. Paired t-test and analysis of variance were conducted to measure the significance of beta diversity at P < 0.05. To evaluate dietary diversity relationships amongst HEC areas, a heatmap was constructed using 1000 bootstrap replications of Bray–Curtis measurements. A Venn diagram was created to determine the shared and unique OTUs between distinct environmental parameters of HEC areas at 97% similarity.

## Data resources

Faecal samples of *E.maximus* were provided by the Wildlife Genetic Resource Bank (WGRB) of PERHILITAN, who collected them from various localities in Peninsular Malaysia. All samples from Kuala Koh, Kenyir, Ulu Muda and Belum-Temenggor were collected by the Management and Ecology of Malaysian Elephants, University of Nottingham Malaysia. Samples were also obtained from HEC locations, such as logged forests, non-logged forests, highways, human settlements, near human settlements etc. Captive samples from the National Elephant Conservation Centre in Kuala Gandah, Pahang served as positive controls and baseline data for the diet of *E.maximus*. A total of 39 feacal samples were utilised, of which 33 were retrieved from 11 HEC areas and six were from captivity (Table 1). During sampling, fresh feacal samples were preserved in 99.9% ethanol prior to laboratory processing. At the National Wildlife Forensic Laboratory (NWFL) of PERHILITAN, samples were kept in a refrigerator at 4℃ for DNA extraction (Syed-Shabthar et al. 2013).

All next-generation sequence data were deposited into National Center of Biotechnology Information (NCBI), under Sequence Read Archive (SRA) accession numbers; SRR19811599, SRR19806293, SRR19806081, SRR19806065, SRR19805810, SRR19805808, SRR19805784, SRR19805749, SRR19805748, SRR19804224, SRR19801341.

## Results

**NGS data analysis**

The concentration of the DNA extracted ranged from 3.2 ng/µl to 385.7 ng/µl. The quantity of DNA measured by the quality check assay was between 3.75 pM to 7.1 pM. High-throughput DNA metabarcoding was used to assess the specific plants consumed from different HEC locations. Illumina NGS successfully produced 379,580 reads, ranging from 4,865 to 99,383 sequences, which were filtered to exclude low-quality sequence reads and chimeras. Subsequently, the OTUs were clustered and 5,385 known OTUs were identified at the 97% similarity cut-off, with the highest in captivity (980) followed by Belum-Temenggor (923) and Kampung Pagi (835) (Table 2). Additionally, 1,563 unique OTUs were found. Table 3 illustrates the grouping of the eleven pooled samples to four main categories of environmental parameters at the studied HEC areas: logged forests (LF), non-logged forests (NLF), human settlements (HS) and human trails (HT). LF had the highest number of plant sequences (118,866), while the most significant and unique OTUs were recorded in HT, followed by LF, NLF and HS (Table 4).

**Plant species identification**

**Figure 2.**
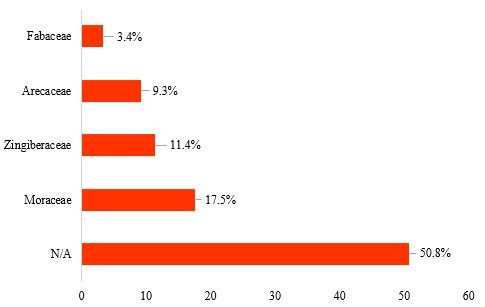
Relative abundance (%) of plants consumed by *E.maximus* at the family level (> 3.4% abundance).

**Figure 3.**
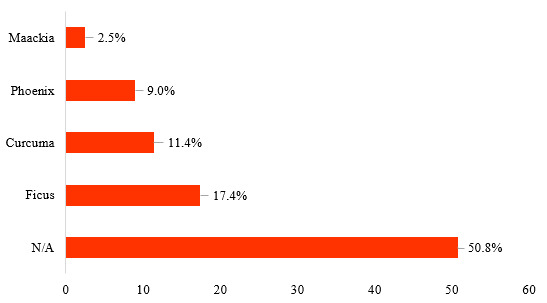
Relative abundance (%) of plants consumed by *E.maximus* at the genus level (> 2.5% abundance).

**Figure 4.**
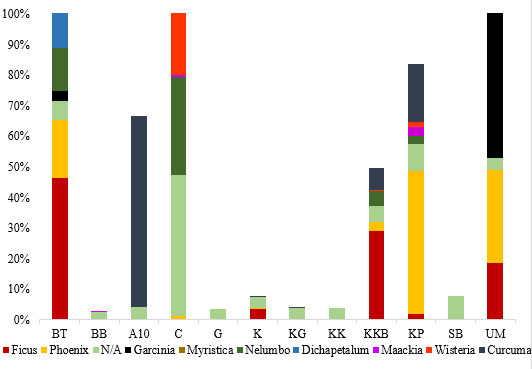
Distribution (%) of plants consumed by *E.maximus* at the genus level (20 most abundant genera). (KK = Kuala Koh; KG = Kupang-Grik; K = Kenyir; KP = Kg. Pagi; KKB = Kg. Kuala Balah; G = Grik; BT = Belum-Temenggor; BB = Bumbun Tahan; UM = Ulu Muda; A10 = Aring 10; SB = Sira Batu; C = Captive; N/A = Not Available).

**Figure 5.**
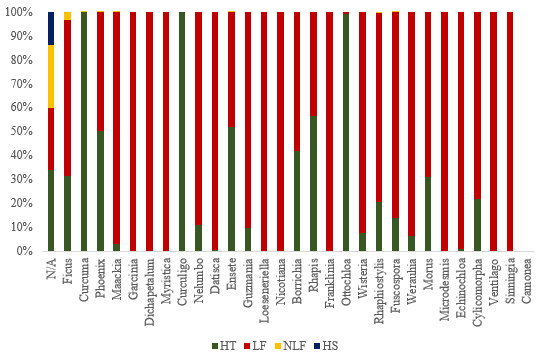
Distribution (%) of plants consumed by *E.maximus* at different environmental parameters at the genus level (20 most abundant genera) (HS = human settlement; LF = logged forest; NLF = non-logged forest; HT = human trail; N/A = Not Available).

The plants consumed by all *E.maximus* sampled in this study were taxonomically classified into 35 orders, 88 families, 196 genera and 237 species (Table 5). Figs 2, 3 depict the relative abundance of dietary plants at the family and genus level in all *E.maximus* samples from HEC locations. Overall, plants belonging to unknown families and genera (N/A) could not be identified by the database and they were the most abundant (50.8%). At the family level, Moraceae (17.5%), Zingiberaceae (11.4%), Arecaceae (9.3%) and Fabaceae (3.4%) predominated in the diet of Asian elephants (Fig. 2). Proportionately, the abundant plant genera were *Ficus* (17.4%), *Curcuma* (11.4%), *Phoenix* (9.0%) and *Maackia* (2.5%) (Fig. 3). Figs 4, 5 highlight the 20 most prominent plants at the genus level in *E.maximus* diets. Kampung Pagi (KP), Belum-Temenggor (BT), Kampung Kuala Balah (KKB) and Ulu Muda (UM) were the HEC areas that covered most of the 20 prominent plant genera (Fig. 4). In contrast, Bumbun Tahan (BB), Grik (G), Kupang-Grik (KG), Kenyir (K), Kuala Koh (KK) and Sira Batu (SB) showed a relatively minimal percentage of discovered genera. LF had the highest abundance of the top 30 plant genera consumed by the wild elephants (Fig. 5).

**Alpha diversity indices, rarefaction curve, heatmap and Venn diagram**

The alpha diversity (Shannon and Chao-1 indices) indicated that the diet of Asian elephants varied depending on the HEC localities (Table 6). KP showed the highest plant diversity with a Shannon index *H* = 2.983, followed by C (*H* = 2.811), KG (*H* = 2.763) and UM (*H* = 2.730). KP also demonstrated significantly high species richness with the greatest Chao-1 value (1,212). BT had a higher Chao-1 value (1,056) than C (1,045). Table 7 shows the alpha diversity, through Shannon and Chao-1 indices, in the diet of wild Asian elephants sorted by the environmental parameters at HEC locations. LF had the highest Shannon index (*H* = 3.033), followed by HT (*H* = 3.006) and NLF (*H* = 2.400). HT had the highest plant richness with a Chao-1 value of 1,870 compared with LF (1,551) and NLF (614.8). The environmental parameter of HS showed the lowest Shannon index (*H* = 2.020) and Chao-1 value (427.6) (Table 7). Table 8 validates the diet of *E.maximus* through paired t-test diversity statistical analysis, based on the Shannon indices. Every pairing of the environmental parameters has a significant P–value defined as P < 0.05 (Table 8). Tables 9, 10 show percentage of plants relative abundance of family and genus consumed by elephants (> 0.1% relative abundance). Table 11 indicates the OTU and percentage of plants eaten at different environmental parameters.

**Figure 6.**
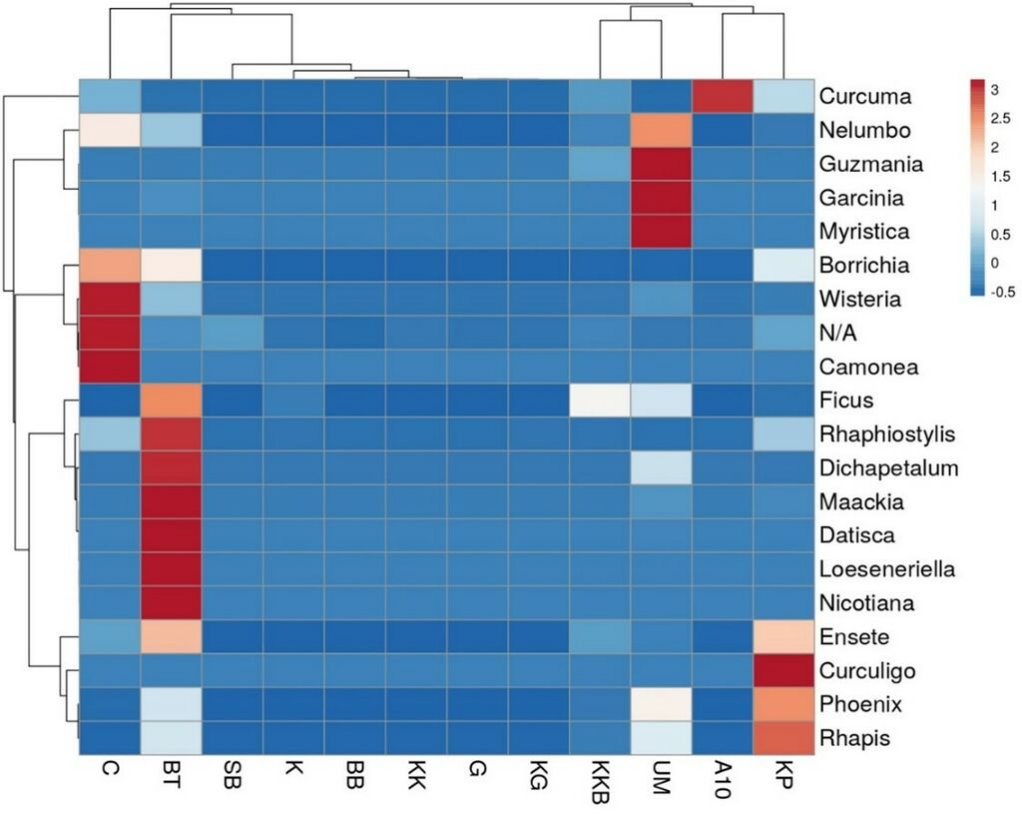
Heatmap with dendrogram showing dietary plant abundance at the genus level for *E.maximus* in different HEC localities. Gradient of the heatmap shows the 20 most abundant genera. KK = Kuala Koh; KG = Kupang-Grik; K = Kenyir; KP = Kg. Pagi; KKB = Kg. Kuala Balah; G = Grik; BT = Belum-Temenggor; BB = Bumbun Tahan; UM = Ulu Muda; A10 = Aring 10; SB = Sira Batu; C = Captive; N/A = Not Available.

**Figure 7.**
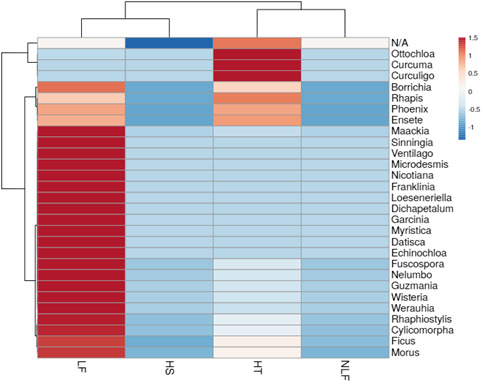
Heatmap with dendrogram showing dietary plant abundance at the genus level for *E.maximus* in different environmental parameters. Gradient of the heatmap shows the 30 most abundant genera. HS = human settlement; LF = logged forest; NLF = non-logged forest; HT = human trail; N/A = Not Available.

**Figure 8.**
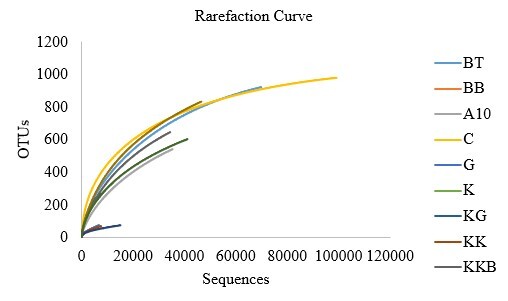
Rarefaction curves for all *E.maximus* samples.

**Figure 9.**
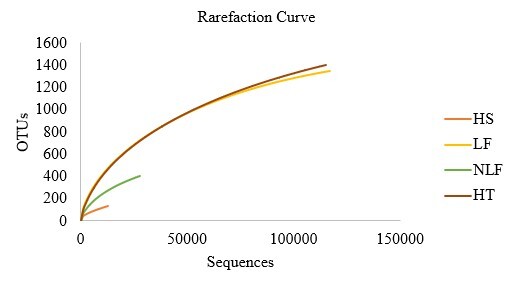
Rarefaction curves for *E.maximus* samples in different environmental parameters at HEC locations.

Figs 6, 7 show the most abundant plant genera consumed by *E.maximus* as a heatmap. The darker the red color, the more prominent the genus was in the elephant diet. *Curculigo*, *Nicotiana*, *Camonea*, *Myristica*, *Garcinia*, *Guzmania*, *Loeseneriella*, *Datisca*, *Wisteria*, *Maackia* and *Curcuma* were the most abundant plant genera identified (Fig. 6). BT contained substantially more dominant genera compared with other HEC localities and among the environmental parameters, LF showed the greatest consumption of plant genera, followed by HT (Fig. 7). Figs 8, 9 show the rarefaction curves between the number of sequences and OTUs, plotted with the help of the rbcL gene metabarcoding database. All rarefaction curves illustrate an increasing pattern where additional sampling needed to be conducted as the plant richness was not sufficiently sequenced. The difference in curves could be affected by the samples or sequencing quality.

**Figure 10.**
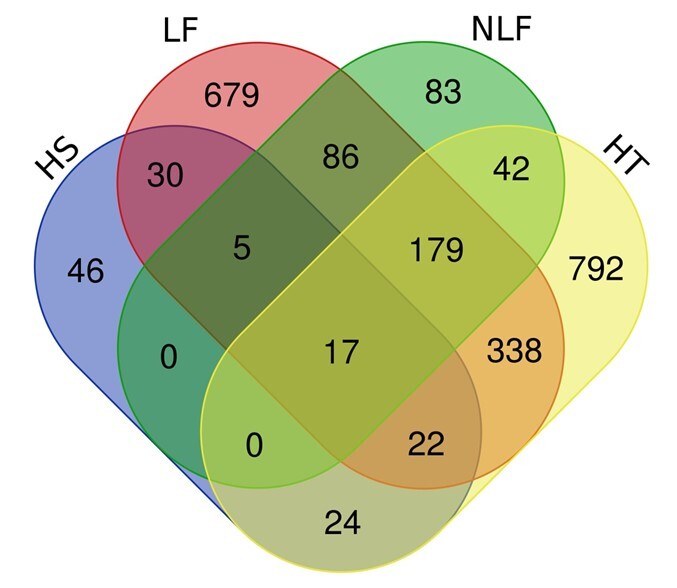
Venn diagram showing the number of shared OTUs amongst environmental parameters in HEC areas at 97% similarity.

**Figure 11.**
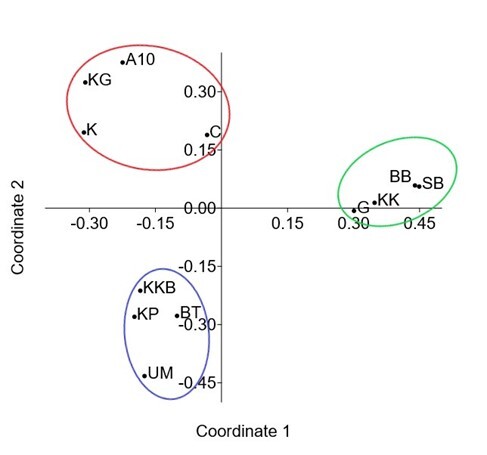
Principal coordinate analysis (PCoA) between Asian elephant samples from different HEC areas, based on Bray–Curtis distances. KK = Kuala Koh; KG = Kupang-Grik; K = Kenyir; KP = Kg. Pagi; KKB = Kg. Kuala Balah; G = Grik; BT = Belum-Temenggor; BB = Bumbun Tahan; UM = Ulu Muda; A10 = Aring 10; SB = Sira Batu; C = Captive.

**Figure 12.**
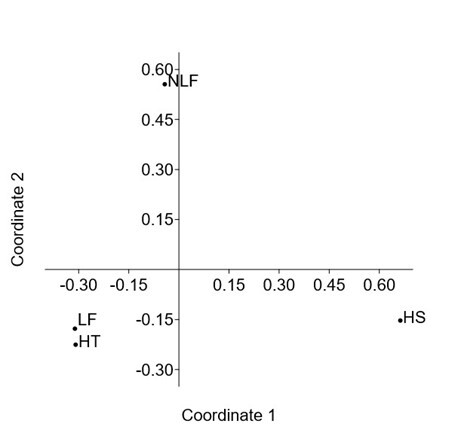
Principal Coordinate Analysis (PCoA) between Asian elephant samples from distinct environmental parameters in HEC areas based on Bray–Curtis distances. HS = human settlement; LF = logged forest; NLF = non-logged forest; HT = human trail.

The Venn diagram in Fig. 10 portrays 1,414 OTUs identified in HT, 1,356 in LF, 412 in NLF and 144 in HS. HT had the highest unique OTUs (792), followed by LF (679), NLF (83) and HS (46). A total of 17 OTUs were shared amongst all environmental parameters in HEC areas (Fig. 10). In this study, PCoA was used to establish the relationship between *E.maximus* samples. Figs 11, 12 demonstrate the grouping of samples by the similarities in feeding patterns. Three clusters of samples from HEC locations were formed: A10–K–KG–C, KP–KKB–UM–BT and BB–SB–G–KK. Samples grouped at smaller distances indicate lesser plant variations amongst the areas involved (Fig. 11). Wild Asian elephant samples from the environmental parameter HP–DM had the same feeding pattern with relatively low plant variations (Fig. 12).

## Discussion

Metabarcoding analysis proved that areas affected by HEC are very attractive to wild Asian elephants. In this study, we examined the diet of wild *E.maximus* from various HEC locations throughout Peninsular Malaysia, without any direct observation of the plants consumed. Previous studies have relied on indirect observations of feeding, including elephant footprints, fresh dung piles near browsed foliage and typical plant damage caused by elephant browsing, such as debarkation, branch breaking and uprooting (English et al. 2014, Koirala et al. 2016, Yamamoto-Ebina et al. 2016). A non-invasive approach using NGS is particularly important for investigating diets of *E.maximus* found in HEC areas, where gaining observational data is difficult. Therefore, this study used a high-throughput DNA metabarcoding approach, targeting the rbcL region to examine the specific plants consumed. To the best of our knowledge, this is the first study using metabarcoding to identify the diet of wild, free-roaming *E.maximus* in HEC areas. These preliminary findings have many crucial implications for mitigation of HEC and the conservation of Asian elephants and other endangered herbivores.

According to our findings, the diversity and richness of the dietary plant taxa are correlated to the quality of an elephant’s habitat (F = 2.159, *P* = 0.014). Alpha diversity analysis demonstrated that KP presented the highest plant diversity, with a Shannon index value of 2.983 and a significantly superior species richness, with a Chao-1 value of 1,212 (Table 6). The diet of wild Asian elephants at KP included the top 20 most abundant plant genera. In a previous study, Jambari et al. (2019) showed that the population of *E.maximus* is dominant in the lowland area of Taman Negara National Park (TNNP). Individual samples from KP originated from HT in TNNP, spanning an area of 4,343 km^2^, with the largest wild Asian elephant population in the world (Karuppannan et al. 2020, Saaban et al. 2020). These animals often choose habitats with abundant food sources, like primary rainforests (Evans et al. 2018). TNNP is now recognised by UNESCO (2021) as a tropical rainforest that is rich in native plants because it has more than 3000 plant species.

The most dominant plant genus *Ficus* (family Moraceae) is the elephant’s main diet preference at BT, UM and KKB. It is a good source of nutrition for fruit-eating animals like *E.maximus* in tropical areas. Figs are rich in fibres, trace minerals, antioxidant polyphenols, proteins, sugars, organic acids, cholesterol-free and contain high number of amino acids (Ercisli et al. 2012, Yemis et al. 2012, Trad et al. 2013, Bey et al. 2013). *Ficus* is native to the eastern Mediterranean region and southwest Asia, being abundantly distributed in primary and secondary forest vegetation (Berg et al. 2006). Accordingly, *Ficus* are found at LF of BT and UM, including HT of KKB at TNNP. The *Ficus* species identified in this study include *Ficus* sp., *F.pandurata*, *F.palmata*, *F.religiosa*, *F.sagittate* and *F.fulva*. Kamaruddin et al. (2019) mentioned that figs cultivated in the open field need a proper management because their growth might easily be affected by environmental factors. Mendoza-Castillo et al. (2016) highlighted that high yield of figs at open field plantations need implementation of fertigation techniques, high planting densities plantation, managing productive branches including macro tunnels and management in handling pruning of leaves, buds and stems.

The genus *Curcuma* (family Zingiberaceae) is the second highest abundant plant genus consumed by wild Asian elephants in the HT of A10, KP and KKB at TNNP. It consists of rhizomatous herbs, such as ginger and turmeric, which are distributed in the tropical and subtropical regions of Southeast Asia, Papua New Guinea and northern Australia (Larsen and Larsen 2006, Kamazeri et al. 2012, Akarchariya et al. 2017). From the rbcL metabarcoding database, *C.aeruginosa*, *C.zedoaria*, *C.longa*, *C.aromatica* Salisb., *G.curtisii* and *C.amada* were detected in the diet of wild *E.maximus*. Yamamoto-Ebina et al. (2016) mentioned that gingers, palms, woody debris and woody fibres are preferred by Asian elephants in both primary and logged forest habitats. Gingers and turmerics tend to grow on forest floors with sunlight exposure in the primary forests of A10, KP and KKB (Evans et al. 2018). Zingiberaceae is commonly identified as one of the main diets of Asian elephants (Chen et al. 2006, English et al. 2014, Yamamoto-Ebina et al. 2016).

Secondary forests and areas with disturbed vegetation have been shown to attract wild Asian elephants and cause HEC (English et al. 2014, De la Torre et al. 2021). In this study, BT recorded the highest number of sequences, known OTUs and unique OTUs, followed by KP and UM. BT showed a considerably high species richness with a Chao-1 value of 1,056 (Table 6). The Belum-Temenggor Forest Complex is one of the tropical lowlands and hill dipterocarp rainforests covering an area of 3,385 km^2^ (Rayan et al. 2012, Rayan and Linkie 2015). Secondary forests with dense vegetation, like BT and UM, encompass the top 20 most abundant plant genera consumed by *E.maximus*. At > 0.1% relative abundance, there are 22 families and 29 genera of dietary plants that could be planted as buffer zones of at least two kilometres from HEC areas (Tables 9, 10). As an introductory, this study emphasises the list of plants up to family and genus levels to ensure accuracy as previous studies only presented the diet of Asian elephants at family level (Sukumar 2003, Chen et al. 2006, English et al. 2014, Koirala et al. 2016, Yamamoto-Ebina et al. 2016, Bahar et al. 2018).

On the other hand, KG, KK and G contained only a small percentage of the top 20 abundant plant genera. Besides grasses, samples retrieved from KG consisted of palm plants, like *Elaeisoleifera* and *Burretiokentiahapala*, which indicates the presence of palm plants near the highway. Wong et al. (2018) emphasised that *E.maximus* living near the Grik-Jeli highway obtain a basic food diet of grasses. The fecal samples from the LF of KG were found along the Kupang-Grik highway, consistent with the fact that elephants are easily attracted to the edges (Campos-Arceiz 2013) and they look for food beside the highway. The plants identified from the samples of KK and G in HS mainly belonged to unknown genera; yet, the low density of plants in these regions suggests that the elephants might be starved for food. Development of smart and green infrastructures, such as ecological corridors with food choices for wildlife including elephants that facilitate their movement from one forested area to another is needed to mitigate HEC cases better in the future (Saaban et al. 2021).

Metabarcoding analysis followed by *t*-test and analysis of variance revealed significant differences in the diets of wild Asian elephants according to environmental parameters (F = 3.002, *P* = 0.029). Elephants were attracted to the diversity of plants in disturbed vegetation locations, such as LF (H = 3.033). In Peninsular Malaysia, secondary forests are areas that are highly suitable for elephant habitats (English et al. 2014, De la Torre et al. 2021). According to Bahar et al. (2018), secondary forests provide many food options for Asian elephants, like wild bananas, sugarcane and palms. This is consistent with our metabarcoding analysis, which shows that the LF have plants such as, *Ensete* (wild banana) and *Phoenix* (palm) in the top 20 most abundantly consumed genera (Fig. 5). LF have the highest number of dietary plant genera, including *Ficus*, *Garcinia*, *Dichapetalum*, *Guzmania*, *Werauhia*, *Wisteria*, *Loeseneriella*, *Nicotiana*, *Fuscospora*, *Rhaphiostylis*, *Myristica*, *Nelumbo* and *Datisca* (*Fig. 7*). Most of the genera listed are included in the top 30 plants genera identified according to distinct environmental parameters of HEC areas studied (Table 11).

Feacal samples were collected from HT of three HEC sites in the primary rainforests of TNNP, namely KP, KKB and A10. HT presented the highest species richness (Chao-1 = 1,870) and a significant diversity index (H = 3.006). Vegetation in these regions showed the maximum number of OTUs (1,414) and unique OTUs (792) compared with other environmental parameters studied. The dominant plant genera in HT were *Curcuma*, *Ottochloa* and *Curculigo* (Fig. 5). The shrubs and grasses found in HT are nourished by photosynthesis that takes place on the forest floor in open canopy areas (Evans et al. 2018). Elephants move within safe habitats and easily avoid disturbances, such as HS, because the environment in protected primary forests has dense vegetation (Talukdar et al. 2020). The paired t-test showed that LF–HT had a significant difference in the identified plant taxa (*P* = 0.003). The grouping of LF–HT samples in PCoA revealed similar dietary patterns with low but significant in terms of plant variations (*P* > 0.05) (Fig. 12).

Feacal samples from NLF and HS mostly comprised unidentified plants (Fig. 7). Metabarcoding analysis of samples from both these environmental parameters revealed only a slight abundance of plant genera, indicating that wild elephants in such areas have limited food resources (Fig. 7). The pair of NLF–HS significantly differed in plant taxa as identified through paired t-tests (*P* = 4.00E-108). Great distance between NLF–HS in the PCoA implied different nutritional patterns with plant variations (*P* > 0.05) (Fig. 12). Evans et al. (2018) stated that elephants in forests that are less dense and have the vegetation of lowlands will find food sources on the outer edge of the forest and hence, are vulnerable to poachers. Wild elephants easily find food and adapt to a forest filled with shrubs and grass-sized plants (English et al. 2014). In a study on forest replanting, plants up to 13 metres high make the most optimal forest statures according to the suitability of elephant habitat (Evans et al. 2018). Information on the diet of wild Asian elephants in different environmental parameters can be used by PERHILITAN as baseline data for conservation planning of *E.maximus* in Peninsular Malaysia.

Even though 50% of the plant families and genera could not be identified by the rbcL metabarcoding database, this study managed to list up to 237 plant species. The most abundant among them were: fig, *Ficus* sp. (17.4%); date palm, *Phoenixdactylifera* (9.0%); black ginger, *Curcumaaeruginosa* (8.0%); white turmeric, *Curcumazedoaria* (2.9%); flowering plants, like *Maackiafloribunda* (2.52%), *Garciniahopii* (0.93%), *Dichapetalumcrassifolium* (0.89%) and *Wisteriafloribunda* (0.81%); and turmeric, *Curcumalonga* (0.51%). Most of the flowering plant species found are not native to Malaysia as the rbcL database tends to identify plants native to African, American and Asian countries. Therefore, the plant species recognised could be close relatives to those in the tropical rainforests of Malaysia (Osman et al. 2020). Common plants detected in the present study that exist in Malaysia include: *Myristicafragrans* (Myristicaceae), *Borrichiafrutescens* (Asteraceae), *Enseteventricosum* (Musaceae), *Ottochloanodosa* (Poaceae), *Echinochloacrus-galli* (Poaceae), *Microdesmescaseariifolia* (Pandaceae), *Imperatacylindrica* (Poaceae), *Sacciolepisindica* (Poaceae), *Musaacuminata* (Musaceae) and *Commelinadiffusa* (Poaceae).

As this was a pilot study, the number of feacal samples examined and HEC areas covered were limited due to the limited availability of stored *E.maximus* feces at WGRB. This study utilised the feces collected by PERHILITAN during opportunistic sampling at HEC areas between 2011 and 2021. Although the poor condition of certain feacal samples may have affected the quality of the extracted DNA or of the sequencing, the rbcL primer intron sequence of all 39 samples was successfully amplified. Prior studies have established that fresh feacal samples lead to high DNA quality and high percentage of sequencing reads (Aifat et al. 2016, Hawlitschek et al. 2018, Karuppannan et al. 2019). We observed this with fresh samples from captivity (C) that displayed a high number of sequences, OTUs unique OTUs and had good plant diversity and richness. Future metagenomics studies using the rbcL region could broaden the plant metabarcoding database to distinguish Malaysian plants more accurately. The results of this study can guide the management of HEC hotspots, thereby influencing the conservation of *E.maximus* to ensure their persistence for a long time in Peninsular Malaysia.

## Conclusions

This study examined plant diversity and richness in the diet of wild *E.maximus* at HEC localities throughout Peninsular Malaysia. DNA metabarcoding using NGS enabled us to identify dietary plant taxa at HEC locations up to the species level. The plant metabarcoding database can be used by PERHILITAN in building buffer zones with plant genera detected in HEC areas through habitat development programs. Future studies with increased periodic sampling at HEC localities are essential to completely understand the dietary diversity of the wild *E.maximus*. Future studies should also include sample of plants damaged by elephants browsing activity in the areas of fecal sample collection to confirm the identification of plant species with voucher samples in Herbarium at Forest Research Institute Malaysia (FRIM). Additional information on fecal freshness level, environmental conditions and surrounding vegetation at sampling sites could improve the quality of findings on metabarcoding diet of wild Asian elephants as well as the habitat enrichment programs within CFS landscape. Conservational efforts to improve the habitat of elephants may mitigate HEC cases and maintain the population of endangered *E.maximus* in Peninsular Malaysia. Metabarcoding using NGS is a useful tool to elucidate the dietary patterns of other significant herbivores to reduce human-wildlife conflicts all around the world.

## Figures and Tables

**Table 1.** List of faecal samples from HEC areas and captivity used in this study.

**No**	**Sample ID**	**Origin**	**Pooled samples**	**Environmental parameters**
1	EM274	Kuala Koh, Kelantan	KK	Near human settlement
2	EM276	Kuala Koh, Kelantan	KK	Near human settlement
3	EM283	Kuala Koh, Kelantan	KK	Near human settlement
4	EM152	Grik, Perak	G	Human settlement
5	EM155	Grik, Perak	G	Human settlement
6	EM159	Grik, Perak	G	Human settlement
7	EM364	Kupang-Grik, Perak	KG	Highway
8	EM365	Kupang-Grik, Perak	KG	Highway
9	EM366	Kupang-Grik, Perak	KG	Highway
10	EM367	Kupang-Grik, Perak	KG	Highway
11	EM368	Kupang-Grik, Perak	KG	Highway
12	EM425	Belum-Temenggor, Perak	BT	Lake side
13	EM426	Belum-Temenggor, Perak	BT	Lake side
14	EM427	Belum-Temenggor, Perak	BT	Lake side
15	EM1363	Ulu Muda, Kedah	UM	Logged forest
16	EM1365	Ulu Muda, Kedah	UM	Logged forest
17	EM1370	Ulu Muda, Kedah	UM	Logged forest
18	EM752	Kg. Pagi, Pahang	KP	Human trail
19	EM755	Kg. Pagi, Pahang	KP	Human trail
20	EM759	Kg. Pagi, Pahang	KP	Human trail
21	EM739	Kg. Kuala Balah, Pahang	KKB	Human trail
22	EM740	Kg. Kuala Balah, Pahang	KKB	Human trail
23	EM668	Aring 10, Pahang	A10	Human trail
24	EM669	Aring 10, Pahang	A10	Human trail
25	EM679	Aring 10, Pahang	A10	Animal trail
26	EM781	Kenyir, Terengganu	K	Non-logged forest
27	EM831	Kenyir, Terengganu	K	Non-logged forest
28	EM832	Kenyir, Terengganu	K	Non-logged forest
29	EM148	Bumbun Tahan. Pahang	BB	Non-logged forest
30	EM149	Bumbun Tahan. Pahang	BB	Non-logged forest
31	EM2167	Sira Batu, Kedah	SB	Non-logged forest
32	EM2168	Sira Batu, Kedah	SB	Non-logged forest
33	EM2169	Sira Batu, Kedah	SB	Non-logged forest
34	EM1524	National Elephant Conservation Centre (NECC), Pahang	C	Captivity
35	EM1531	National Elephant Conservation Centre (NECC), Pahang	C	Captivity
36	EM1548	National Elephant Conservation Centre (NECC), Pahang	C	Captivity
37	EM1541	National Elephant Conservation Centre (NECC), Pahang	C	Captivity
38	EM1527	National Elephant Conservation Centre (NECC), Pahang	C	Captivity
39	EM1537	National Elephant Conservation Centre (NECC), Pahang	C	Captivity

**Table 2.** Number of sequences, OTUs and unique OTUs of plants consumed by *E.maximus*.

**Samples**	**Sequences**	**OTUs**	**Unique OTUs**
KK	7,769	71	17
KG	7,448	272	55
K	9,574	294	48
KP	46,757	835	176
KKB	34,625	648	175
G	6,967	78	19
BT	70,022	923	360
BB	4,865	60	12
UM	41,396	605	152
SB	15,248	76	19
A10	35,526	543	85
C	99,383	980	445
Total	379,580	5385	1563

**Table 3.** List of pooled samples according to distinct environmental parameters of all HEC areas.

No	Pooled Samples	Environmental Parameters
1	Kuala Koh, Kelantan (KK)	Human Settlements (HS)
2	Grik, Perak (G)	
3	Belum-Temenggor, Perak (BT)	Logged Forests (LF)
4	Kupang-Grik, Perak (KG)	
5	Ulu Muda, Kedah (UM)	
6	Bumbun Tahan, Pahang (BB)	Non-logged Forests (NLF)
7	Kenyir, Terengganu (K)	
8	Sira Batu, Kedah (SB)	
9	Aring 10, Pahang (A10)	Human Trails (HT)
10	Kg. Kuala Balah, Pahang (KKB)	
11	Kg. Pagi, Pahang (KP)	

**Table 4.** Number of sequences, OTUs and unique OTUs of plants eaten by elephants at different environmental parameters of HEC areas (HS = human settlement; LF = logged forest; NLF = non-logged forest; HT = human trail).

Samples	Sequences	OTUs	Unique OTUs
HS	14,736	144	46
LF	118,866	1,356	679
NLF	29,687	412	83
HT	116,908	1,414	792
Total	280,197	3,326	1,600

**Table 5.** Taxonomic classification of plants consumed by *E.maximus* according to rbcL gene analysis.

**Taxonomic level**	**Total number**
Order	35
Family	88
Genus	196
Species	237

**Table 6.** Alpha diversity indices of Shannon and Chao-1 values for *E.maximus*.

**Samples**	**Shannon_H**	**Chao-1**
KK	1.112	141
KG	2.763	326.3
K	2.58	411.8
KP	2.983	1,212
KKB	2.267	968.9
G	1.806	303.6
BT	2.702	1,056
BB	1.162	93.83
UM	2.730	913.4
SB	0.746	193.2
A10	1.998	828.3
C	2.811	1,045

**Table 7.** Alpha diversity indices of Shannon and Chao-1 values at different environmental parameters in HEC areas.

Samples	Shannon_H	Chao-1
HS	2.020	427.6
LF	3.033	1,551
NLF	2.400	614.8
HT	3.006	1,870

**Table 8.** Paired t-test diversity statistical analysis of plant diets in different environmental parameters, based on Shannon indices (HS = human settlement; LF = logged forest; NLF = non-logged forest; HT = human trail).

Pairing	t	df	p-value
LF–HS	-69.212	23029	0
LF–NLF	-48.916	52632	0
LF–HT	-2.9376	2.34E+05	0.003
HT–NLF	-47.93	48619	0
HT–HS	-68.583	21555	0
HS–NLF	-22.167	34775	4.00E-108

**Table 9.** Percentage of plants relative abundance consumed by Asian elephants at studied HEC areas at family level (N/A = not available) (> 0.1% abundance).

No	Family	BT	BB	A10	C	G	K	KG	KK	KKB	KP	SB	UM	Total
1	N/A	6.21	2.52	3.97	45.84	3.61	3.68	3.82	4.03	5.45	8.89	7.91	4.07	50.77
2	Moraceae	46.39	0.00	0.02	0.10	0.00	3.61	0.00	0.00	29.19	1.98	0.00	18.71	17.52
3	Zingiberaceae	0.23	0.00	62.36	11.01	0.00	0.00	0.01	0.00	7.45	18.92	0.00	0.02	11.41
4	Arecaceae	18.95	0.00	0.14	1.34	0.00	0.14	0.03	0.00	2.83	46.62	0.00	29.95	9.25
5	Fabaceae	72.27	0.03	0.02	18.54	0.00	0.05	0.02	0.00	0.09	2.75	0.00	6.22	3.40
6	Clusiaceae	3.25	0.00	0.03	0.03	0.00	0.00	0.00	0.00	0.03	0.00	0.00	96.67	0.93
7	Dichapetalaceae	76.96	0.00	0.03	0.00	0.00	0.00	0.00	0.00	0.03	0.00	0.00	22.98	0.89
8	Nelumbonaceae	14.15	0.00	0.00	32.16	0.00	0.00	0.00	0.00	4.65	2.72	0.00	46.31	0.53
9	Bromeliaceae	1.03	0.00	0.05	0.59	0.00	0.00	0.00	0.00	6.81	2.22	0.00	89.30	0.49
10	Myristicaceae	0.00	0.00	0.00	0.00	0.00	0.00	0.00	0.00	0.06	0.00	0.00	99.94	0.47
11	Hypoxidaceae	0.17	0.00	0.62	0.11	0.00	0.00	0.00	0.00	0.17	98.70	0.00	0.23	0.46
12	Poaceae	23.10	0.00	43.13	30.06	0.00	0.00	0.00	0.00	1.31	2.17	0.00	0.23	0.46
13	Asteraceae	32.01	0.00	0.27	44.34	0.00	0.07	0.00	0.00	0.20	22.64	0.00	0.47	0.39
14	Datiscaceae	98.32	0.00	0.00	0.23	0.00	0.00	0.00	0.00	0.00	0.46	0.00	1.00	0.34
15	Musaceae	38.80	0.00	2.97	8.27	0.00	0.08	0.00	0.00	8.11	37.83	0.00	3.94	0.33
16	Convolvulaceae	0.62	0.00	0.00	98.75	0.00	0.18	0.00	0.00	0.09	0.09	0.00	0.27	0.30
17	Celastraceae	99.80	0.00	0.00	0.00	0.00	0.00	0.00	0.00	0.10	0.00	0.00	0.10	0.26
18	Solanaceae	100.00	0.00	0.00	0.00	0.00	0.00	0.00	0.00	0.00	0.00	0.00	0.00	0.25
19	Metteniusaceae	66.50	0.12	0.12	14.96	0.00	0.49	0.49	0.00	0.62	16.56	0.00	0.12	0.21
20	Theaceae	99.87	0.00	0.00	0.00	0.00	0.00	0.00	0.00	0.00	0.00	0.00	0.13	0.20
21	Nothofagaceae	28.53	0.00	0.00	3.57	0.00	0.16	0.00	0.00	6.05	8.37	0.00	53.33	0.17
22	Pandaceae	66.75	0.00	0.00	0.00	0.00	0.00	0.00	0.00	0.00	0.00	0.00	33.25	0.11

**Table 10.** Percentage of plants relative abundance consumed by Asian elephants at studied HEC areas at genus level (N/A = not available) (> 0.1% abundance).

No.	Genus	BT	BB	A10	C	G	K	KG	KK	KKB	KP	SB	UM	Total
1	N/A	6.21	2.52	3.97	45.84	3.61	3.68	3.82	4.03	5.45	8.89	7.91	4.07	50.77
2	*Ficus*	46.39	0.00	0.02	0.10	0.00	3.64	0.00	0.00	29.21	1.96	0.00	18.68	17.39
3	*Curcuma*	0.21	0.00	62.39	11.02	0.00	0.00	0.01	0.00	7.45	18.90	0.00	0.02	11.39
4	*Phoenix*	18.97	0.00	0.13	1.32	0.00	0.14	0.02	0.00	2.62	46.58	0.00	30.21	8.98
5	*Maackia*	90.40	0.04	0.03	0.44	0.00	0.07	0.02	0.00	0.05	2.91	0.00	6.03	2.52
6	*Garcinia*	3.25	0.00	0.03	0.03	0.00	0.00	0.00	0.00	0.03	0.00	0.00	96.66	0.93
7	*Dichapetalum*	76.96	0.00	0.03	0.00	0.00	0.00	0.00	0.00	0.03	0.00	0.00	22.98	0.89
8	*Wisteria*	15.46	0.00	0.00	76.25	0.00	0.00	0.00	0.00	0.23	1.59	0.00	6.47	0.81
9	*Nelumbo*	14.15	0.00	0.00	32.16	0.00	0.00	0.00	0.00	4.65	2.72	0.00	46.31	0.53
10	*Myristica*	0.00	0.00	0.00	0.00	0.00	0.00	0.00	0.00	0.06	0.00	0.00	99.94	0.47
11	*Curculigo*	0.00	0.00	0.63	0.11	0.00	0.00	0.00	0.00	0.17	99.09	0.00	0.00	0.46
12	*Borrichia*	31.81	0.00	0.27	44.66	0.00	0.00	0.00	0.00	0.21	22.71	0.00	0.34	0.39
13	*Datisca*	98.32	0.00	0.00	0.23	0.00	0.00	0.00	0.00	0.00	0.46	0.00	1.00	0.34
14	*Ensete*	39.98	0.00	1.08	8.36	0.00	0.08	0.00	0.00	7.78	38.66	0.00	4.06	0.32
15	*Camonea*	0.00	0.00	0.00	100.00	0.00	0.00	0.00	0.00	0.00	0.00	0.00	0.00	0.29
16	*Guzmania*	0.09	0.00	0.09	0.37	0.00	0.00	0.00	0.00	9.59	0.00	0.00	89.86	0.29
17	*Loeseneriella*	99.79	0.00	0.00	0.00	0.00	0.00	0.00	0.00	0.10	0.00	0.00	0.10	0.26
18	*Nicotiana*	100.00	0.00	0.00	0.00	0.00	0.00	0.00	0.00	0.00	0.00	0.00	0.00	0.24
19	*Rhaphiostylis*	66.50	0.12	0.12	14.96	0.00	0.49	0.49	0.00	0.62	16.56	0.00	0.12	0.21
20	*Ottochloa*	0.51	0.00	93.13	4.96	0.00	0.00	0.00	0.00	0.64	0.25	0.00	0.51	0.21
21	*Rhapis*	20.52	0.00	0.00	0.00	0.00	0.00	0.00	0.00	3.12	53.25	0.00	23.12	0.20
22	*Franklinia*	99.87	0.00	0.00	0.00	0.00	0.00	0.00	0.00	0.00	0.00	0.00	0.13	0.20
23	*Fuscospora*	28.31	0.00	0.00	3.60	0.00	0.16	0.00	0.00	5.56	7.86	0.00	54.50	0.16
24	*Werauhia*	2.18	0.00	0.00	0.54	0.00	0.00	0.00	0.00	0.91	5.44	0.00	90.93	0.15
25	*Morus*	45.73	0.00	0.00	0.21	0.00	0.00	0.00	0.00	26.07	4.70	0.00	23.29	0.12
26	*Echinochloa*	91.76	0.00	0.23	7.55	0.00	0.00	0.00	0.00	0.23	0.23	0.00	0.00	0.12
27	*Microdesmis*	66.58	0.00	0.00	0.00	0.00	0.00	0.00	0.00	0.00	0.00	0.00	33.42	0.11
28	*Cylicomorpha*	64.64	0.00	0.00	0.00	0.00	0.00	0.00	0.00	0.00	21.64	0.00	13.72	0.10
29	*Ventilago*	100.00	0.00	0.00	0.00	0.00	0.00	0.00	0.00	0.00	0.00	0.00	0.00	0.10

**Table 11.** OTU and percentage of plants plants eaten by elephants at different environmental parameters of HEC areas at top 30 genus level (N/A = not available) (HS = human settlement; LF = logged forest; NLF = non- logged forest; HT = human trail).

No.	Genus	DM	%		HP	%		HTP	%		PM	%
1	N/A	35 285		30.42	27 180		23.27	27 187		91.67	14 721	100.00
2	*Ficus*	20 588		17.75	42 955		36.78	2402		8.10	0	0.00
3	*Curcuma*	38 371		33.08	103		0.09	2		0.01	0	0.00
4	*Phoenix*	16 816		14.50	16 773		14.36	50		0.17	0	0.00
5	*Maackia*	286		0.25	9211		7.89	11		0.04	0	0.00
6	*Garcinia*	2		0.00	3532		3.02	0		0.00	0	0.00
7	*Dichapetalum*	2		0.00	3392		2.90	0		0.00	0	0.00
8	*Myristica*	1		0.00	1781		1.52	0		0.00	0	0.00
9	*Curculigo*	1749		1.51	0		0.00	0		0.00	0	0.00
10	*Nelumbo*	149		0.13	1222		1.05	0		0.00	0	0.00
11	*Datisca*	6		0.01	1297		1.11	0		0.00	0	0.00
12	*Ensete*	574		0.49	532		0.46	1		0.00	0	0.00
13	*Guzmania*	105		0.09	976		0.84	0		0.00	0	0.00
14	*Loeseneriella*	1		0.00	967		0.83	0		0.00	0	0.00
15	*Nicotiana*	0		0.00	924		0.79	0		0.00	0	0.00
16	*Borrichia*	339		0.29	470		0.40	0		0.00	0	0.00
17	*Rhapis*	434		0.37	336		0.29	0		0.00	0	0.00
18	*Franklinia*	0		0.00	768		0.66	0		0.00	0	0.00
19	*Ottochloa*	739		0.64	8		0.01	0		0.00	0	0.00
20	*Wisteria*	56		0.05	675		0.58	0		0.00	0	0.00
21	*Rhaphiostylis*	140		0.12	543		0.46	5		0.02	0	0.00
22	*Fuscospora*	82		0.07	506		0.43	1		0.00	0	0.00
23	*Werauhia*	35		0.03	513		0.44	0		0.00	0	0.00
24	*Morus*	144		0.12	323		0.28	0		0.00	0	0.00
25	*Microdesmis*	0		0.00	407		0.35	0		0.00	0	0.00
26	*Echinochloa*	3		0.00	401		0.34	0		0.00	0	0.00
27	*Cylicomorpha*	82		0.07	297		0.25	0		0.00	0	0.00
28	*Ventilago*	0		0.00	377		0.32	0		0.00	0	0.00
29	*Sinningia*	0		0.00	332		0.28	0		0.00	0	0.00
30	*Camonea*	0		0.00	0		0.00	0		0.00	0	0.00
